# Human immunodeficiency virus positive status disclosure among children in northwest Ethiopia: a cross-sectional study

**DOI:** 10.4314/ahs.v23i1.20

**Published:** 2023-03

**Authors:** Getachew Asmare Adella, Meklit Abie Yimer, Endeshaw Chekol Abebe

**Affiliations:** 1 School of Public Health, College of Health Science and Medicine, Wolaita Sodo University, Wolaita Sodo, Ethiopia, Ethiopia; 2 College of Health Sciences, Debre Tabor University, Debre Tabor, Ethiopia; 3 Reproductive Health and Nutrition Department, School of Public Health College of Health Science and Medicine, Wolaita Sodo University, Wolaita Sodo, Ethiopia

**Keywords:** ART, Bahir Dar, Caregiver/mother, Children, HIV, HIV positive status disclosure

## Abstract

**Background:**

Human Immunodeficiency Virus positive status disclosure is an essential component of pediatric care and longterm disease management. However, one of the greatest challenges that caregiver/mothers and health care workers face is the disclosure of HIV positive status to children living with the virus. Therefore, the objective of this study was to assess HIV positive status disclosure and associated factors among HIV positive children in northwest Ethiopia.

**Methods:**

Institutional based cross-sectional study was conducted among 417 sampled HIV positive children attending pediatric ART clinics of public health facilities from February 01 to March 30 2020 in northwest Ethiopia. Simple random sampling technique was used to select study participants. A structured interviewer administered questionnaire was used for data collection and the collected data entered into Epi data software. Binary logistic regression analysis was done and variables with P-value <0.05 was considered as a significant predictors of HIV positive status disclosure of HIV positive children.

**Result:**

From 417 sampled population, 390 were involved in this study making 93.5% response rate. The study revealed that 53.6% with 95% CI (0.486-0.586) of HIV positive children knew their HIV positive sero status. Caregiver/mothers who had greater than three family sizes (AOR=1.984, 95% CI=1.046-3.762), children whose ages greater than 10 years (AOR=6.679, 95% CI=3.372-13.227) and children on ART for more than 5 years (AOR=8.96, 95% CI=6.402-12.257) were predictors of HIV positive status disclosure.

**Conclusion:**

The HIV positive status disclosure was high in the study area relative to other studies. Family size, children age, and length of children on ART were predictors of HIV positive status disclosure for HIV positive children. Health care providers, especially those working at pediatrics ART clinics should keep these factors in mid while working with caregivers to encourage disclosure of HIV positive status.

## Introduction

Pediatric human immunodeficiency virus (HIV) infection remains a major issue worldwide. According to UNAIDS 2021 report, globally about 1.2-2.2 million with an average of 1.7 million children younger than 15 years are living with HIV. Among these 100,000-240,000 with average of 150,000 children are new HIV infections. AIDS related deaths among children were 68,000-160,000 with average of 99,000^1^. Globally many children with HIV(CHIV) are surviving into adolescence as a result of increased access to antiretroviral therapy (ART) with children and adolescents living longer with HIV on ART the focus turns from survival to improving quality of life, treatment adherence, retention in care and treatment, viral suppression and sustaining physical and mental well-being 2. ART prevents an estimated 4.2 million deaths in low- and middle-income countries in 2002–2012 and the number of children who are younger than fifteen, receiving ART has increased from 566,000 in 2011 to 630,000 in 2012 3.

Pediatrics HIV positive status disclosure means telling the children that they have HIV 4,5. The depth of HIV positive status information to be shared with children the manner and time of disclosure are things to be considered by caregiver/mothers and healthcare workers 6. American Academy of Pediatrics and WHO strongly recommend a gradual process of giving age-appropriate information to HIV infected school-age children by considering the child's cognitive and emotional development. i.e., b/c they must be fully informed in order to appreciate consequences for many aspects of their health, including sexual behavior and treatment decisions to be supportive and non-judgmental 4, 7, 8.

Disclosure is one of the greatest challenges that families and health care providers face to decide how and when to disclose HIV positive status to children 2. Caregiver/mothers frequently experience uncertainty in revealing an HIV positive status to their children which stems from fears of negative consequences from disclosure such as psychological problems, inability to comprehend and deal with the diagnosis, stigma, discrimination and unintended disclosure to others 9. However, HIV/AIDS disclosure becomes more significant because of the multiple benefits for the children and their caregiver/mothers 10. Disclosure helps create a sense of closeness in the family, reduce feelings of anxiety and isolation on the part of the parents/caregiver/mother. It also relieves the burden of living with the secret of being HIV-positive, build social support networks 7.

Studies indicated the high rates of delayed disclosure or non-disclosure among HIV-positive children yet how and when caregiver/mothers and healthcare professionals disclose to children are not well-characterized and the number of children that know their status is generally thought to be low 5. HIV disclosure practices in sub-Saharan African countries remain complex due to the immense influence of politics, culture and HIV surveillance limitation 11.

Studies done in Ethiopia showed that the magnitude of HIV positive status disclosure ranges from 17.4% - 49.4%^12^–^19^. It is important to know the proportion of disclosure and its associated factors in the study area to design an appropriate intervention contributing towards achieving the HSTP of three 90's, especially the third 90% (suppressing the viral load by 90% among ART started) and SDG. Therefore, the study aimed to assess HIV positive status disclosure and associated factors among HIV positive children in pediatric ART clinics of public health facilities in northwest Ethiopia.

## Methods and materials

### Study area and period

The study was conducted from February 01 to March 30 2020 in pediatric ART clinics in Bahir Dar city public health facilities Northwest Ethiopia. Bahir Dar is the capital city of Amhara regional state and far from 480 km from Addis Ababa. The city has nine public health facilities (three hospitals and six health centers) of the two hospitals (Felege Hiwot Referral and Addis Alem primary hospitals) and five health centers (Bahir Dar, Han, Abay, shim bit and Dagmawi Minilic) provides ART service. Currently 12570 clients were on ART follow-up in the seven ART sites. From the total ART clients, pediatric client accounts 663 among these 620 were in the age group of 7-15 years 20.

### Study design

An institutional based cross-sectional study design was used.

### Source population

The source populations were all HIV positive children who have regular ART follow up in pediatric ART clinics in Bahir Dar city public health facilities.

### Study population

The study populations were all HIV positive children age from 7-15 years who have ART follow up in pediatric ART clinics in Bahir Dar city public health facilities.

### Inclusion and exclusion criteria

The study included caregiver/mothers of HIV positive children age from 7-15 years who attending pediatric ART clinics and excluded street children who come without caregiver/mothers and caregiver/mothers who have HIV positive children who are unable to respond during data collection.

### Sample size determination and sampling procedure

The sample size was determined by using single population proportion formula based on the following assumptions: 95 % level of confidence, the proportion of 44% (the proportion of HIV positive disclosure among HIV positive children in Gondar town public health facilities in 2018 (12) and 5% margin of error.

n= ((Z α /2)2 p (1-P)) / d2

n=the required sample size

p=the proportion of HIV positive disclosure among HIV infected children

Z α/2=the critical value at 95% confidence level (1.96).

d=the margin of error between the sample and the population =5%

n=1.96x1.96x.44x.56/ (0.05x0.05)

n=379 considering 10% of non-response rate, n=417

The sampling techniques were first all seven-government health facilities providing pediatric ART and care service in the city were included in the study. Second, the sampling frame (list of HIV positive children of those ages from 7-15 years and currently on ART follow-up) was obtained from recorded computer data. Third, the number of respondents to be included in the study, sample was determined proportionally following the total number of children. Fourthly, simple random sampling with a table of random numbers was applied to select respondents. Children who became alone for ART follow up without their caregiver/mothers during data collection were appointed for the other day to contact their caregiver/mothers to collect data.

### Dependent variable

HIV positive status disclosure of HIV positive children

### Independent variables

Sociodemographic factors of caregiver/mothers and children, sociocultural and personal factors of caregiver/mothers and children, clinical factors of caregiver/mothers and children, and service /program related factors.

### Data collection tools and procedures

A structured interviewer-administered a pre-tested questionnaire, document review and observation was used. The questionnaire was developed in the English language that includes all the relevant variables to meet the objectives (16-19). Then it was translated into the Amharic language for better understanding and to make it easy for data collection as the study area uses Amharic language and finally translate the Amharic version back to English to check for its original meaning. A total of four diploma nurses were allocated for data collection and two-degree nurses to supervise the data collectors from out of the working place in Bahir Dar city health institutions. During data collection, the respondents were asked individually with a face-to-face interview in a separate room.

### Data quality control

For data quality control purpose, the data collectors and supervisors were trained before the data collection. Supervision was done during the data collection period and the questionnaires were pretested. The pre-test was done on (eighteen caregiver/mothers of HIV positive children) aged 7-15 years in Zenzelma health center a week before data collection.

### Operational and term definitions

**Pediatrics HIV positive status Disclosure:** -telling for children that they have HIV regardless of who told the children 3, 4.

**Primary caregivers:** - an adult aged ≥18 years who lives with the child, participates in the child's daily care and who knows most about the child's health 16–19.

**Biological parent:** -a person he/she who has a blood relationship with the child or who born the child 16–19.

**Stigma and Discrimination:** a child is stigmatized or discriminated against if a parent/caregiver answers above the three of the stigma and discrimination questions 16–19

**Knowledge of caregivers about disclosure:** a caregiver/parent was considered as knowledgeable if he /she answer above the three of the knowledge questions 16–19.

**The attitude of caregivers towards disclosure:** a caregiver/parent has a favourable attitude towards disclosure if he/she answers above the four of the attitude questions^16^–^19^.

### Data processing and analysis

Data were edited, cleaned, coded, and entered to Epi-Data version 3.1 and then was exported to SPSS version 25.0 for analysis. Descriptive statistics were done by computing summary statistics like frequency, mean, percentages, and standard deviations, and the results were presented in tables and graphs. Binary logistic regression analysis was done. All variables having a P ≤ of 0.2 in bivariate analysis were considered as a candidate for multivariable logistic regression to control for possible confounding effects. Multivariable logistic regression was applied to see the independent effect of each variable on the outcome variable. Multi-collinearity among the independent variables was checked using VIF and Hosmer and Lemeshow test was used to assess models Goodness of fit (72.1%). Final results of association were presented based on adjusted Odds Ratio at 95% confidence level and p< 0.05 was considered statistically significant.

### Ethical approval and consent to the participant

Ethical approval was obtained from the Institutional Review Board (IRB) of Bahir Dar University College of Medicine and Health Sciences. A permission letter was obtained from Bahir Dar University College of Medicine and Health Sciences. The consent form was read to the participants and written consent was obtained from each caregiver/mother before the interview. Participants were informed as they can skip question/s that they don't want to answer partially or fully and also to stop at any time if they want to do so. Confidentiality of the individual information was assured by not recording the identifying information.

## Result

### Socio-demographic characteristics of caregiver/mothers and children

A total of 390 caregiver/mothers of HIV positive children were interviewed make a response rate of 93.5%. Of the total caregiver/mothers who participated in study 221 (54.1%) were between the age groups of 31-40 years. The mean age of respondents was (39.8±7.25). The majority of respondents were orthodox Christian followers 352(90.3%). Two hundred twenty-nine (58.7%) of the caregiver/mothers were married. The majority of caregiver/mothers 318(81.5%) were females. The employment status of 100(25.6%) of caregiver/mothers was daily laborers. Three hundred seventy-eights (96.9%) were Amhara by ethnicity. Two hundred four (52.3%) of the caregiver/mothers/parents had less than three children. Among family income 276(70.8%) of respondents had >1000-birr monthly income. The socio-demographic characteristics of the child 261(66.9%) of them were less than ten years and with mean, median, range of 11.38,11 and 7-15 respectively. Two hundred thirteen (54.6%) of them were males and 347 (89%) children were in primary school (Table 1).

**Table 1:** Socio-demographic characteristics of caregiver/mothers and children among HIV positive children in pediatric ART clinics of public health facilities in northwest Ethiopia in 2020 (n=390)

Variables	Category	Frequency/percent
Age	<30	54 (13.3%)
	31–40	221(54.1%)
	41–50	99 (25.4%)
	51–60	26 (6.7%)
Sex	Female	318 (81.5%)
	Male	72 (18.5%)
Religion	Orthodox	352 (90.3%)
	Muslim	26 (6.7%)
	Protestant	12 (3.1%)
Level of education	Unable to read and write	84 (21.5%)
	Able to read and write only	124 (31.8%)
	primary education (1–8)	60 (15.4%)
	Secondary education and above	122 (31.3%)
Ethnicity	Amhara	378 (96.9%)
	Oromo	7 (1.8%)
	Tigre	5 (1.3%)
Marital status	Married	229 (58.7%)
	Single	13 (3.3%)
	Widowed	92 (23.6%)
	Divorced	56 (14.4%)
occupation	Unemployed	100 (25.6%)
	Daily labourer	100 (25.6%)
	Government employ	64 (16.4%)
	Private employ	88 (22.6%)
	Merchant	38 (9.7%)
Total monthly income	<500	21 (5.4%)
	501–1000	93 (23.8%)
	>1000	276 (70.8%)
Total family size	<3	204 (52.3%)
	≥3	186 (47.7%)
Age of the child	< 10 years	261 (66.9%)
	≥ 10 years	129 (33.1%)
Sex of the child	Female	177 (45.4%)
	Male	213 (54.6%)
School grade of the child	Not started education	3 (0.8%)
	KG	2 (0.5%)
	Primary education	347(89%)
	Secondary education	38 (9.7%)

### Socio-cultural characteristics of caregiver/mothers and children

One hundred twenty-five (32%) were living with their mothers and fathers and 158(40.5%) of them lost their family members due to HIV among these 75(19.2%) were lost their mothers. Three hundred sixteen (81%) of children had biological parents and 232(59.5%) of them faced stigma due to their HIV status (Table 2).

**Table 2:** Socio-cultural characteristics of caregiver/mothers and children among HIV positive children in pediatric ART clinics of public health facilities in northwest Ethiopia, 2020

Variables	Categories	Frequency/percent
With whom the child currently live	Mother	156 (40%)
	Father	21(5.4%)
	Both mother and father	125(32%)
	Relatives	88(22.6%)
A child lost any of family member Due to HIV?	Yes	158 (40.5%)
	No	232(59.5%)
Who was lost?	Mother only	75(19.2%)
	Father only	35(9%)
	Siblings	5(1.3%)
	Both mother and Father	43(11%)
Relationship to the child?	Biological parent	316(81%)
	Grandparent	31(7.9%)
	Siblings	17(4.4%)
	Relatives	26(6.7%)
Stigma due to HIV	yes	232(59.5%)
	No	158(40.5%)

### Knowledge and Attitude of caregiver/mothers about HIV status disclosure

About two hundred eighteen (55.9%) of respondents had a favourable attitude towards HIV positive status disclosure. Three hundred eight (79 %) of caregiver/mothers were knowledgeable about HIV positive status disclosure.

### Clinical characteristics of caregiver/mothers and children

Three hundred twenty-one (82.3%) of the caregiver/mothers were HIV positive. Of them, all were on ART. Two hundred sixty-one (66.9%) of children had been diagnosed to have HIV below five years and 129 (33.1%) of them diagnosed above five years. Three hundred forty-six (88.7%) of the children WHO clinical stage was stage I and all of the children start ART at the time of the survey. Two hundred fifty-eight (66.2%) of the child start ART at age below five years and 238(61%) of the child took ART for more than five years. Three hundred sixty-eight (94.4%) of the children were good treatment adherence and 102(26.2%) of them had ever been affected with opportunistic infection (Table 3).

**Table 3:** Clinical characteristics of caregiver/mothers and children for HIV positive status disclosure among HIV positive children in pediatric ART clinics of public health facilities in northwest Ethiopia, 2020 (n=390)

Variables	Categories	Frequencies/percentage
HIV status of the caregiver/mother	Positive	321(82.3%)
	Negative	69(17.7%)
Did the caregiver/mother start ART	Yes	321(100%)
Age HIV positive diagnosed	< 5 years	261(66.9%)
	≥ 5 years	129(33.1%)
WHO clinical stage of the child	Stage I	346(88.7%)
	Stage II	28(7.2%)
	Stage III	16(4.1%)
Age ART initiated	< 5years	258(66.2%)
	≥ 5 years	132(33.8%)
Duration on ART?	< 5 years	152(39%)
	≥ 5 years	238(61%)
Other medication the child took other than ART	Bactrim	99(25.4%)
	Anti-tuberculosis	11(2.8%)
	tuberculosis-prophylaxis	27(6.9%)
Treatment adherence of the child	Good	368(94.4%)
	Fair	22(5.6%)
The child develops opportunistic infections within one year	Yes	102(26.2%)
	No	288(73.8%)

### Service/program related factors

With service or program-related factors, all of the health facilities had a guideline for HIV positive status disclosure and 257(65.9%) of the health, facilities had separated the pediatrics ART clinic.

### HIV positive status disclosure among HIV positive children

In this study 209 (53.6%) with 95% CI (48.6% -58.6%) of HIV positive children knew their HIV positive sero status and 160(76.5) of them knew their HIV positive status at the age from 7-10 years. Seventy-one (34%) and eighty-five 40.7%) of mothers and health care providers disclosed children's HIV positive status respectively. About 181(46.4%) of the children were not disclosed their HIV positive status and the reason told for visiting health facilities was 75(41.4%) for tuberculosis follow-up. From those not disclosed, 106(58.6%) of them had a plan for disclosure in future (Table 4).

**Table 4:** HIV positive status disclosure among HIV positive children in pediatric ART clinics of public health facilities in northwest Ethiopia, 2020

Variables	Categories	Frequency/percent
HIV positive status disclosed (n=390)	Yes	209(53.6%)
	No	181(46.4%)
Age at disclosed(n=209)	7–10	160(76.5%)
	11–15	49(23.5%)
Who disclosed HIV status (n= 209)	Mother	71(34%)
	Father	24(11.5%)
	Grandparents	9(4.3%)
	Siblings	9(4.3%)
	Relatives	11(5.2%)
	Health care providers	85(40.7%)
Reason for visiting a health facility for non-disclosed(n=181)	For tuberculosis follow up	75(41.4%)
	For cardiac follow up	17(9.4%)
	Others	89(49.2%)
Have a plan to disclose(n=181)	Yes	106(58.6%)
	No	75(41.44%)

### Factors affecting for pediatric HIV positive status disclosure

Variables: child lose family member due to HIV, caregiver/mothers' relation to the child, HIV status of the caregiver/mother, facilities having separated pediatrics ART clinic, age of the caregiver/mother, total monthly income of the caregiver/mother, family size, age of the child, stigma and discrimination, attitude towards disclosure, age at diagnosis, age at ART initiation, duration on ART fulfills chi-square assumption and were candidate variables with p<0.2. The Hosmer-Lemeshow goodness-of-fit was 0.721.

Caregivers who had greater than three family size, children aged over 10 years and children who were on ART for more than 5 years were predictors of HIV positive status disclosure among HIV positive children. Caregivers who had greater than three family sizes were two times more odds of disclosing their children's HIV positive status than those with less than three family sizes (AOR=1.984, 95% CI=1.046-3.762). Children whose ages above 10 years were 6.7 times more likelihood to disclose their HIV positive status compared to those aged below 10 years (AOR=6.679, 95% CI=3.372-13.227). Children who were on ART for more than 5 years were nearly nine times more likely to be disclose their HIV positive status than those who were on ART for less than 5 years (AOR=8.96, 95% CI=6.402-12.257). (Table 5).

**Table 5:** Factors affecting HIV positive status disclosure among HIV positive children in pediatric ART clinics of public health facilities in northwest Ethiopia, 2020 (n=390)

Variables	Disclosure status		COR (95% CI)	AOR (95% CI)	p-value
	Yes	No			
**Age of the child**					
< 10 years	26	115	1	1	
≥ 10 years	183	66	**12.264(7.363–20.428)**	**6.679(3.372–13.227)**	**0.000** *
**A child lost any family member due to HIV**					
Yes	94	64	1.494(0.933–2.249)	4.78(0.892–9.362)	0.543
No	115	117	1	1	
**An attitude of caregiver/mothers towards HIV positive status disclosure**					
Favourable attitude	64	154	0.077(0.047–0.128)	2.72(0.803–3.898)	0.126
Unfavourable attitude	145	27	1	1	
**Family size**					
<3	101	103	1	1	
≥3	108	78	1.412(0.946–2.107)	**1.984(1.046–3.762)**	**0.002** *
**Duration of ART**					
<5 years	28	124	1	1	
≥ 5 years	181	57	14.063(8.472–23.342)	**8.96(6.402–12.257)**	**0.000** *

* Statistically significant at p<0.05

## Discussion

The study tried to determine the proportion of disclosure and associated factors among HIV positive children aged 7-15 years. This study revealed that 53.6% (CI (48.6%-58.6%) of HIV positive children knew their sero status. The finding of this study was higher than the study done in Gondar public health facilities, Gondar hospital, Bahir Dar city public health facilities, Addis Ababa, Western Kenya, Tanzania, Zambia, Urban clinics of Kampala Uganda, Tertiary Health Facility in Abuja Nigeria, Papua New Guinea 12, 15, 17, 19, 21–26. The reason for the higher finding in this study is children are engaged in HIV dialog with their parents and family members with important approaches for HIV positive disclosure like increased awareness on the benefit of disclosure for caregiver/mothers and due to favourable attitude. The higher prevalence of disclosure in the study might be due to decreased fear of stigma and discrimination by the family members and the child.

However, the finding of this study was lower as compared to studies conducted in Rwanda is 68% in 2012^27^. This could be due to socio-cultural difference, good child-parent interaction and the presence of better health care services that promote disclosure.

Children whose ages above 10 years were 6.7 times more likelihood to disclose their HIV positive status compared to those aged below 10 years. This finding is consistent with a study done in Gondar public health facilities, Gondar hospital, Jinja hospital in Uganda, Tertiary Health Facility in Abuja Nigeria, and South Africa 11, 12, 17,25, 28. The reason may be the child is matured to understand the illness and may have less chance for the child leaking of the family's secret in a study done in Gondar hospital. Most caregiver/mothers preferred to disclose the HIV status to older children because they believed they would understand the nature of the diagnosis and keep it secret in South Africa.

Children who were on ART for more than 5 years were nearly nine times more likely to be disclose their HIV positive status than those who were on ART for less than 5 years The finding of this study was consistent with the study done in Gondar public health facilities, Bahir Dar and Ghana 17,19,29. This is possibly because when children stay on ART for a longer period of time, they may have more frequent visits to the ART clinic, which leads to caregivers and children having repeated contact with health care providers. As a result, caregivers and children might receive regular counselling, which aids disclosure. Another likely explanation is that children who have been on ART for a long time do not have symptoms. This leads to them to questioning why they are on medication though they are well, which may result in decreased adherence and thus the caregivers' last option to disclose the child's HIV status.

Caregivers who had greater than three family sizes were two times more odds of disclosing their children's HIV positive status than those with less than three family sizes. This study was consistent with the study done in South Africa 30. This could be if the size of the family increases, the fear of HIV transmission to negative siblings and other family members among parents increases, which enforces them to reveal the HIV positive status of their children.

## Limitation of study

The present study has some limitations. Firstly, the study design was cross-sectional, which may not show the temporal relationship between the cause and effect (between HIV positive status disclosure and independent variables). At the same time, since it used a quantitative approach rather than a qualitative approach, it might not explore the possible reasons for non-disclosure, traditional and cultural factors of the participants.

## Conclusion

The HIV positive status disclosure among HIV positive children in the study area was higher compared with other studies done in other areas. Caregiver/mothers who have greater than three family size, caregiver/mothers who have children whose ages greater than 10 years and caregiver/mothers whose children on ART for more than 5 years were predictors of HIV positive status disclosure. HIV care providers should consider these factors while working with caregiver/mothers to encourage disclosure of HIV positive status. For better understanding of reason for non-disclosure and cultural traditional and cultural factors, qualitative study is recommended for future study.

## Data Availability

The datasets used and/or analysed during the current study are available from the corresponding author on reasonable request.

## List of abbreviations

AIDS: Acquired Immune Deficiency Syndrome
AOR: Adjusted Odds Ratio
ART: Anti Retroviral Therapy
CHIV: Children with HIV
CI: Confidence Interval
HIV: Human Immune Virus
UNAIDS: United Nations Aid for International Development
SDG: Sustainable Development Goal
WHO: World Health Organization
